# Defining post–colon capsule endoscopy colorectal cancer (pCCECRC): an International Capsule Endoscopy Research (iCARE) group consensus statement

**DOI:** 10.1007/s00464-026-12892-5

**Published:** 2026-05-27

**Authors:** Hussain Ibrahim, Ian Lei, Peter Murchie, Anastasios Koulaouzidis, Angus Watson, the iCARE consensus collaborative, James Turvill, Sunil Dolwani, Stephan Haas, Artur Nemeth, Ervin Toth, Begoña González Suárez, Lucian Negreanu, Aileen McKinley, Niels Qvist, Foong Way David Tai, Deirdre McNamara, Stephen McSorley, Ioanna Parisi, Faidon-Marios Laskaratos, Ed Seward, Cristiano Spada, Reena Sidhu, Martin Keuchel, John Plevris, Ulrik Deding, Bruno Rosa, Eimear Gibbons, Ruari Jardine, Campbell Macleod, Gemma Mcinally, Gabriele Wurm Johansson, Sergio Cadoni, Renato Cannizzaro, Anirudh Pramod Bhandare, Amit Chattree, Victoria Fawcett, Alexander Robetson, Wojciech Marlicz, Feliz Akyuz, Jack Winter, Fraser Maxwell

**Affiliations:** 1 University of Aberdeen, Aberdeen, UK; 2 NHS Highland, Inverness, UK; 3 University Hospital of Coventry and Warwickshire, Coventry, UK; 4 University of Southern Denmark, Odense, Denmark

**Keywords:** Colon capsule endoscopy, Colorectal cancer, Interval cancers, Quality assurance

## Abstract

**Introduction and aims:**

Colon capsule endoscopy (CCE) has seen increased adoption as a large bowel diagnostic test over the past decade. Unlike colonoscopy, the post-test colorectal cancer (CRC) outcomes are yet to be standardised and reported. This international consensus aimed to establish a framework to define pCCECRC.

**Methods:**

A modified Delphi process was conducted in accordance with CREDES guidance. Expert panellists were recruited from the International Capsule Endoscopy Research (iCARE) group and leads of CCE services in the United Kingdom and Ireland. Statements were informed by a systematic literature review and developed by a core steering group. Two rounds of anonymous online voting were undertaken to measure expert agreement on a total of 11 statements. A scale of 1 (strongly disagree) to 5 (strongly agree) was utilised. Agreement was defined as ≥ 75% agree or strongly agree with a maximum IQR of 1.

**Results:**

40 experts participated in round one, with 36 completing round two. Consensus was achieved for all statements. One statement, relating to how pCCECRC should be defined did not reach the consensus threshold in round 1 and was revised before reaching consensus in round 2. pCCECRC was endorsed as an appropriate quality metric, with definitions aligned to PCCRC where feasible. A 6-month post-procedure grace period was agreed, and reporting of a pCCECRC-3-year rate was recommended. Separate unadjusted (service-level) and adjusted (test-specific) pCCECRC rates were defined. A structured “most plausible explanation” framework for attribution is proposed that both aligns with PCCRC methodology and considers the more complex diagnostic pathway of CCE services that usually entails multiple tests.

**Conclusion:**

This consensus proposes standardised definitions and reporting recommendations for pCCECRC. The proposed framework is intended to support quality assurance and evaluation of CCE services. Validation using real-world service datasets is required.

**Supplementary Information:**

The online version contains supplementary material available at https://doi.org/10.1007/s00464-026-12892-5.

Quality assurance (QA) is central to diagnostic services, providing a structured approach to ensure investigations are delivered consistently, safely, and with acceptable diagnostic accuracy [1]. In colorectal cancer (CRC) diagnostics, QA has been operationalised most extensively for colonoscopy, which remains the reference standard investigation [2]. These metrics evolved in response to accumulating evidence of substantial variation in colonoscopy performance between operators and services [3]. Variation in bowel preparation assessment, caecal intubation, withdrawal technique, and adenoma detection has been shown to translate into differences in subsequent CRC risk [4]. Consequently, international organisations have emphasised routine audit of outcomes and the use of defined performance indicators as core components of colonoscopy QA. One such metric is post-colonoscopy CRC (PCCRC) [2, 5].

PCCRC refers to CRC diagnosed after a colonoscopy in which no malignancy was identified, following an accepted post-procedure grace period. The World Endoscopy Organisation (WEO) recommends reporting PCCRC when cancer is diagnosed between 6 months and 3 years after a ‘clear’ colonoscopy, commonly reported as the PCCRC-3-year rate [6]. In 2016, the British Society of Gastroenterology (BSG) incorporated PCCRC-3-year reporting into minimum QA standards, requiring services to identify these events and maintain systems for their routine audit [2]. Since publication of the WEO consensus, multiple population-based studies demonstrated wide variation in PCCRC-3-year rates across healthcare systems, typically in the range of approximately 5–9% [7–9]. These variations reflect differences in colonoscopy quality, service organisation, and patient pathways. In England, for example, the overall PCCRC between 2005 and 2013 was 7.4%, compared with 3.6% within the Bowel Cancer Screening Program (BCSP), where enhanced endoscopist accreditation and quality oversight are in place [10].

Colon capsule endoscopy (CCE) has been increasingly adopted over the past decade as a minimally invasive alternative diagnostic modality for colonic evaluation. Although it is used far less frequently than colonoscopy, established CCE services now exist in multiple healthcare systems [11–13]. Data from established CCE programmes now provide a basis for service-level evaluation, which was one motivation for developing this consensus framework. Since CCE services are relatively limited compared to colonoscopy, standardised reporting supports comparison between services, shared learning, and future pooled analysis. Like colonoscopy, CCE aims to detect colorectal pathology by visualising the colonic mucosa but differs fundamentally in its mode of delivery and clinical integration [14]. The pill-size capsule relies on gut motility to complete colonic transit within its battery lifespan, with transit commonly augmented by booster agents and, in some protocols, prokinetic medications [15, 16]. To date, post-CCE colorectal cancer (pCCECRC) rates have not been systematically reported at service-level due to the relatively recent scale-up of CCE services and the absence of mature longitudinal follow-up in most cohorts.

The WEO consensus statement on PCCRC includes a framework for post-imaging CRC (PICRC), which in principle could be applied to pCCECRC. Unlike colonoscopy, CCE frequently requires downstream investigations to achieve a definitive diagnosis, making direct application of PCCRC methodology insufficient. Because post-test investigation rates after colonoscopy are low, PCCRC reflects both procedural performance and the diagnostic pathway. CCE is associated with lower examination completion rates and a substantially higher requirement for downstream investigations to achieve definitive diagnosis or to treat identified pathology [11–13]. A single pCCECRC rate may therefore fail to distinguish between the diagnostic performance of CCE and the adequacy of the wider diagnostic pathway.

As CCE services expand, the absence of a shared framework for defining and reporting pCCECRC risks inconsistent interpretation and non-comparable reporting across centres. A structured definition is therefore needed to enable audit, service evaluation, and standardised reporting.

## Methods

A modified Delphi process was conducted in accordance with the ‘Conducting and REporting of DElphi Studies’ (CREDES) reporting standard [17], to develop consensus amongst CCE experts on pCCECRC.

### Systematic review and statement generation

To inform statement development, a systematic literature review was conducted on the 20th of September 2025 covering published literature since 1988. Two separate searches were conducted in the Medline, Embase and Cochrane databases. The search strings are detailed in the supplementary material. The aim of the search was to identify the literature relevant to PCCRC and CRC in the context of CCE. Search outputs were exported to Rayyan (Qatar Computing Research Institute, Qatar). Title and abstract review were undertaken by one author (HI), with full text review undertaken of the eligible studies, or when eligibility was uncertain. Studies not published in English and those without full text were excluded. Evidence reviews and statement generation conducted by the core steering group of authors: HI, AK, IL, PM, and AW. Statements were developed before Round 1, however they were open to alteration based on panellist feedback. Ultimately, one statement was revised, as described in the results section.

### Expert recruitment

This Delphi process was initially advertised at the sixth annual *The Future of Minimally Invasive GI & Capsule Diagnostics* (REFLECT) symposium held in Nyborg, Denmark in September 2025. Attendees were encouraged to take part or express an interest in participating. Experts were then recruited from 2 sources: the iCARE group members mailing list, and by emailing the leads of CCE services in the UK and Ireland to invite participation, with the aim of using a snowballing recruitment model.

Individuals who met at least one of the participation criteria listed in Table 1 were invited to take part. Panellists were specifically asked if they meet at least one of the criteria. Only those who answered ‘yes’ were able to progress to the questionnaire.

**Table 1:** Essential criteria for participation in the Delphi process

Lead/founder of a CCE service
Certified CCE reader (average > 50 reads/year over the past 2 years)
≥ 5 peer-reviewed CCE papers (past 2 yrs, 1st/2nd/senior author)
Emerging experts who have led innovative CCE research but do not meet the above thresholds

Three members of the steering group were also voting members of the panel. The recommendations of this process are aimed at CCE service providers. Due to the nature of the recommendations, wider inclusion of lay persons and medical professionals, that do not have CCE experience, was not deemed appropriate since a good understanding of the challenges facing CCE is required. CCE use is, however, relatively new and not as widespread as other diagnostic modalities, and therefore has a limited pool of experts. We considered 15 panellists per round adequate to reach a stable estimate and aimed to recruit a minimum of 20 to account for potential attrition [18]. This study was reviewed by the University of Aberdeen Ethics Review Board and was deemed exempt from formal ethical approval. Participants were informed about the study rationale and process in the invitation email and on the welcome page of the questionnaire. Consent to participate was implied through completion of the questionnaire.

### Measuring agreement

Panellists were asked to rank statements on a Likert scale of 1 (strongly disagree) to 5 (strongly agree). Consensus criteria were defined in advance. Agreement was measured using the percentage of panellists who agree (4) or strongly agree (5) with the proposed statements. Additionally, the degree of dispersion was assessed using the interquartile range (IQR) of Likert scores. Consensus was considered reached if ≥ 75% agreed with a statement with a maximum IQR of 1.

### Conducting the survey

We aimed to conduct the survey over 2 rounds. In round 1, the panellists voted on the definition of pCCECRC. In the subsequent round we aimed to ask the panellists about the aetiological and attribution nomenclature of pCCECRC. Any items that failed to reach consensus were reviewed by the working group, amended as appropriate and re-introduced in the next round, with the introduction of further rounds if deemed necessary. The Delphi process was stopped when consensus was reached on all items, or when there was stability across rounds. Written feedback was provided to panellists both in the invitation email and in the welcome message before the second round. The survey was conducted using a dedicated online survey platform, Welphi (Portugal). The software allowed all participants to vote on items anonymously, with identities hidden from the working group.

## Results

### Panellists

Forty panellists participated in round 1, of whom 36 (90%) completed round 2. Participating panellists voted on all items in each round, with no missing items. Responses from panellists who did not participate in round 2 were retained for analysis of round 1 statements. Analyses were conducted using available responses for each round. All panellists were based in Europe, with the majority practising in the United Kingdom (21/40). Other countries represented included Denmark (4), Sweden (4), Italy (3), Ireland (2), and six additional European countries (one panellist each). Most panellists were gastroenterologists (31/40), with additional representation from colorectal surgery (6), epidemiology (1), advanced nursing practice (1), and clinical endoscopy (1). Most reported substantial experience with CCE, with 24 reporting at least five years’ experience and a further 12 reporting three or more years of involvement. Nearly all panellists had extensive colonoscopy experience, with 37 reporting > 5 years in practice. Annual CCE reporting volumes varied, with approximately half of panellists reporting > 50 cases/year. Seven panellists reported no annual CCE volume and two had no clinical CCE experience. This reflects the inclusion of CCE researchers under criterion 3 of the eligibility criteria. Details are displayed in Table 2.

**Table 2:** Breakdown of panellist country of practice, speciality, and colonoscopy and CCE experience

Country of practice	
United Kingdom	21
Denmark	4
Sweden	4
Italy	3
Ireland	2
Portugal, Poland, Germany, Türkiye, Romania, Spain	1 from each
Speciality	
Gastroenterologist	31
Surgeon	6
Epidemiologist	1
Colorectal nurse practitioner	1
Clinical endoscopist	1
Years of experience with CCE (requesting, reporting, interpreting)	
5 or more years	24
3–4 years	8
1–2 years	4
< 1 year	2
None	2
Years of colonoscopy experience	
5 or more years	37
1–2 years	2
None	1
Approximate annual CCE volume	
> 100	13
51–100	7
25–50	9
1–25	4
None	7

### Consensus statements

Consensus was reached for all proposed statements following the Delphi process. One statement was revised after round 1 and achieved consensus in round 2. Agreement levels for all statements are detailed in Table 3.

**Table 3:** Delphi statements and agreement levels amongst panellists

Statement	% agreement	Median Likert score (IQR)
1. pCCECRC is an appropriate quality metric for CCE services	86%	4 (4–5)
2. pCCECRC definitions should align with post-colonoscopy CRC	93%	5 (4–5)
3. In keeping with colonoscopy methodology, a 6-month timeframe/grace period from the date of the CCE procedure is appropriate for the CCE pathway to achieve a colorectal cancer diagnosis	94%	4 (4–5)
4. A service-level "unadjusted pCCECRC" rate is necessary for pathway evaluation	90%	4 (4–5)
5. A test-specific "adjusted pCCECRC" rate is necessary for CCE diagnostic accuracy	88%	4 (4–5)
6. Both the unadjusted and adjusted pCCECRC rates should ideally be reported separately for quality assurance and test evaluation purposes	93%	4 (4–5)
7. The pCCECRC-3y (CRC diagnosed 6 months to 3 years after going through the CCE pathway where no CRC was detected) should be reported by CCE services as a measure of the diagnostic service's ability to detect and prevent CRC: $\frac{{{\text{CRC detected 6 months}}\, - \,{\text{3years after CCE where no CRC was detected }}\left( {{\mathrm{pCCECRC}}} \right)}}{{{\text{CRC detected within 6 months of CCE }}\left( {{\mathrm{Detected}}} \right){\text{ + CRC detected 6 months}}\, - \,{\text{3years after CCE where no CRC was detected }}\left( {{\mathrm{pCCECRC}}} \right)}}$	94%	4 (4–5)
8. The CCE pathway may involve multiple tests to achieve CRC diagnosis, adding complexity when determining the aetiology of pCCECRC. We therefore suggest extending the use of "most plausible explanation" from PCCRC terminology when describing the aetiology of pCCECRC	97%	4 (4–4)
9. To facilitate the use of a common language with colonoscopy services, unadjusted pCCECRCs can be categorised as follows: - A: Possible missed lesion, prior examination adequate. This can be further categorised into: - A1: attributed to adequate CCE - A2: attributed to adequate colonoscopy/FSIG/CTC - B: Possible missed lesion, prior examination negative but inadequate - C: Detected lesion, not resected - D: Likely incomplete resection of previously identified lesion OR - Likely new CRC—for cancers detected 4 years following CCE As per colonoscopy methodology, the modifying statement “deviation from the planned management pathway” is added to the above categories when appropriate	97%	4 (4–5)
10. To facilitate categorisation of pCCECRC by most plausible explanation, we recommend CCE reporting adheres to the standard previously established by the Nyborg consensus statement on unifying terminology, reporting, and bowel preparation standards in CCE	100%	5 (4–5)
11. Certain aspects of PCCRC nomenclature and recommendations are not specific to colonoscopy, and their use should be extended to pCCECRC. These include: - Sub-categorisation of post-test cancers into interval vs non-interval–to ensure service adherence to recommended surveillance intervals - The minimum histological and molecular features data of CRC should be documented as per the WEO consensus statement on PCCRC	97%	4 (4–5)

*Statement 1: pCCECRC is an appropriate quality metric for CCE services.*

CCE is increasingly used as a diagnostic alternative to colonoscopy but remains characterised by lower procedural volumes, variable completion rates, and a higher requirement for downstream investigations. It does however provide a more comfortable patient experience and increases diagnostic capacity, which is the rationale for ongoing use [19, 20]. Unlike colonoscopy, established performance indicators and outcome-based QA metrics for CCE are limited. As a diagnostic test intended to detect CRC and advanced neoplasia, evaluation of post-test CRC outcomes is therefore relevant to both test performance and service delivery. Available evidence indicates that CCE demonstrates good sensitivity for colorectal neoplasia, particularly for larger lesions [21]. In this context, pCCECRC represents a potential QA indicator that captures not only test performance but also pathway execution, including adequacy of follow-up and timeliness of diagnosis.

*Statement 2: pCCECRC definitions should align with post-colonoscopy CRC.*

Both CCE and colonoscopy are used to detect colorectal cancer and may be associated with post-test cancer diagnoses [22]. Where methodological differences permit, alignment of pCCECRC definitions with established PCCRC terminology supports internal comparison within healthcare systems and facilitates consistent attribution of post-test cancer aetiology. Equally, quality improvement and any lessons learned can apply across both diagnostic services.

*Statement 3: In keeping with colonoscopy methodology, a 6-month timeframe/grace period from the date of the CCE procedure is appropriate for the CCE pathway to achieve a colorectal cancer diagnosis.*

An initial statement proposing a 6-month grace period for completion of the CCE diagnostic pathway did not reach consensus, prompting revision to clarify that the interval commenced from the date of the CCE examination rather than referral. Following this clarification, consensus was achieved that a 6-month post-procedure grace period is appropriate to allow completion of diagnostic investigations in more complex pathways. In keeping with established PCCRC methodology, CRCs diagnosed within this interval were considered detected by the diagnostic service. Although panellists were not required to explain their disagreement with the initial statement, it is likely that the statement was rejected because it departed from the PCCRC convention, which defines post-test cancers as those diagnosed more than 6 months after a colonoscopy in which no cancer was detected.

*Statement 4: A service-level “unadjusted pCCECRC” rate is necessary for pathway evaluation.*

The panel agreed that a service-level pCCECRC rate, incorporating all patients entering the CCE diagnostic pathway regardless of examination completeness or subsequent investigations, is necessary to evaluate overall pathway performance. This unadjusted rate reflects the ability of a diagnostic service to achieve timely CRC diagnosis across the full pathway, including follow-up investigations prompted by CCE findings or technical limitations.

*Statement 5: A test-specific “adjusted pCCECRC” rate is necessary for CCE diagnostic accuracy.*

The panel agreed that a test-specific pCCECRC rate restricted to CCE examinations that were considered to be complete and adequately cleansed at the time of reporting is required to evaluate intrinsic test performance. This adjusted rate isolates diagnostic accuracy by excluding cases in which cancer diagnosis was influenced primarily by examination incompleteness or downstream investigations, allowing focussed assessment of lesion detection and reporting during CCE.

*Statement 6: Both the unadjusted and adjusted pCCECRC rates should ideally be reported separately for quality assurance and test evaluation purposes*

The panel agreed that reporting both unadjusted and adjusted pCCECRC rates is necessary because they address different aspects of performance within the CCE diagnostic pathway. The unadjusted pCCECRC rate captures outcomes for all patients entering the pathway, regardless of examination completeness or need for downstream investigation, and therefore reflects overall service-level performance and pathway execution. In contrast, the adjusted pCCECRC rate is restricted to complete and adequately cleansed examinations and is intended to assess intrinsic test performance, independent of pathway-related factors. Reporting these rates separately avoids ambiguity in case inclusion and supports clearer interpretation of both service delivery and diagnostic accuracy.

*Statement 7: The pCCECRC-3y (CRC diagnosed 6 months to 3 years after going through the CCE pathway where no CRC was detected) should be reported by CCE services as a measure of the diagnostic service's ability to detect and prevent CRC*

$$\frac{{{\text{CRC detected 6 months}}\,{-}\,{\text{3years after CCE where no CRC was detected }}\left( {{\mathrm{pCCECRC}}} \right)}}{{{\text{CRC detected within 6 months of CCE }}\left( {{\mathrm{Detected}}} \right){\text{ + CRC detected 6 months}}\,{-}\,{\text{3years after CCE where no CRC was detected }}\left( {{\mathrm{pCCECRC}}} \right)}}$$

The panel agreed that a pCCECRC-3-year rate, defined as CRC diagnosed between 6 months and 3 years after completion of the CCE diagnostic pathway in cases where no cancer was initially detected, should be reported by CCE services. In keeping with established PCCRC methodology, this rate is calculated as the proportion of post-test cancers diagnosed within the 6-month to 3-year interval, represented by the numerator in the above formula, relative to all CRCs detected either within or after the defined post-procedure grace period. The latter includes all cancers diagnosed following CCE and is represented by the denominator in the above formula. Use of a 3-year reporting interval aligns pCCECRC with existing PCCRC frameworks and supports comparison of diagnostic pathway performance within healthcare systems.

*Statement 8: The CCE pathway may involve multiple tests to* *achieve CRC diagnosis, adding complexity when determining the aetiology of pCCECRC. We therefore suggest extending the use of "most plausible explanation" from PCCRC terminology when describing the aetiology of pCCECRC*

The panel agreed that attribution of post–CCECRC is inherently more complex than after colonoscopy, owing to higher rates of incomplete examinations and the frequent need for downstream investigations to achieve definitive diagnosis. CCE can have a high follow-up investigation rate [23], with up to 60% of patients requiring follow-up investigation in some cohorts [12], In this context, application of a structured “most plausible explanation” framework, adapted from established PCCRC methodology, was considered appropriate to support consistent classification of pCCECRC aetiology whilst acknowledging pathway complexity and the role of clinical judgement. Central or dual clinician review may be necessary to ensure accurate attribution of aetiology in complex pCCECRC cases.

*Statement 9: To facilitate the use of a common language with colonoscopy services, unadjusted pCCECRCs can be categorised as follows:*

- *A: Possible missed lesion, prior examination adequate. This can be further categorised into:*
- *A1: attributed to adequate CCE*
- *A2: attributed to adequate colonoscopy/FSIG/CTC*
- *B: Possible missed lesion, prior examination negative but inadequate*
- *C: Detected lesion, not resected*
- *D: Likely incomplete resection of previously identified lesion*

OR

- *Likely new CRC–for cancers detected 4 years following CCE*

As per colonoscopy methodology, the modifying statement “deviation from the planned management pathway” is added to the above categories when appropriate.

The root causes/most plausible explanation of PCCRC is classified based on whether an adenoma was seen in the bowel segment of interest, and whether it was resected. If no lesion was seen, the adequacy of colonoscopy is considered. This subdivides the aetiology of PCCRC into the following categories:

- A: Possible missed lesion, prior examination adequate.
- B: Possible missed lesion, prior examination negative but inadequate.
- C: Detected lesion, not resected.
- D: Likely incomplete resection of previously identified lesion [6]

**Fig. 1:**
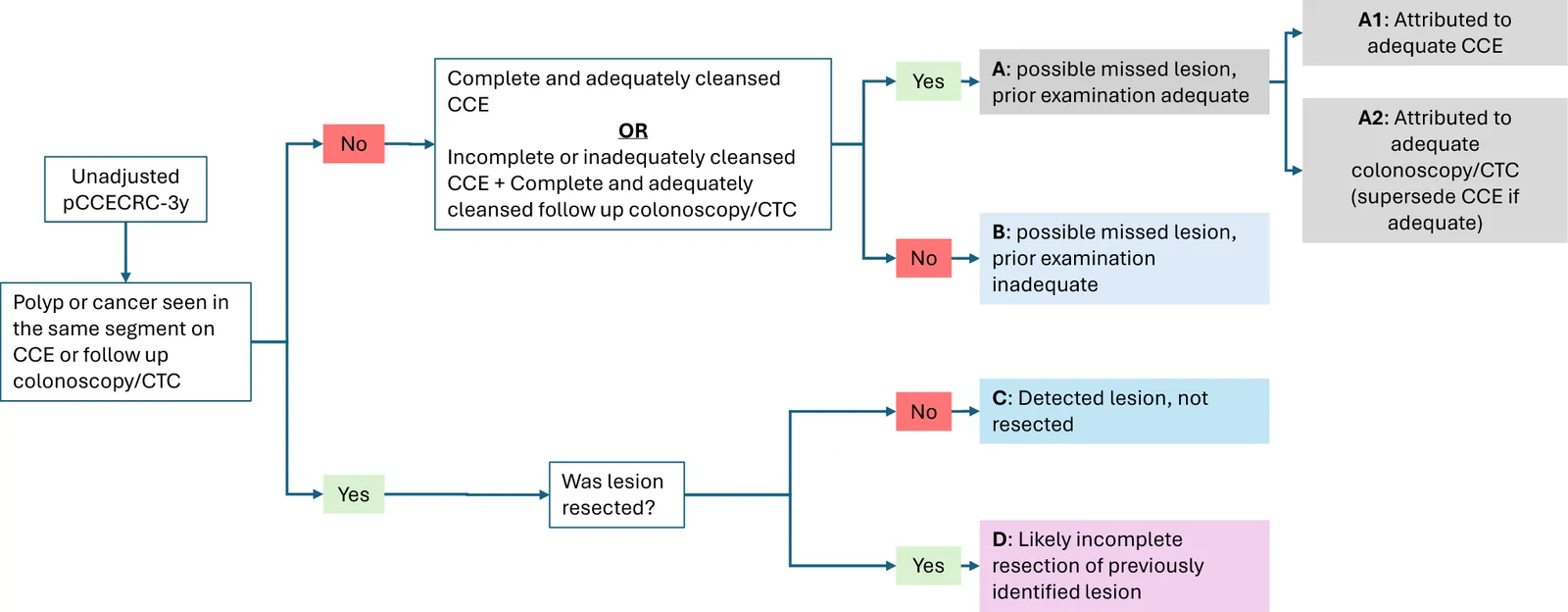
Proposed process for attribution of aetiology/most plausible explanation of post Colon Capsule Endoscopy Colorectal Cancer (pCCECRC). pCCECRC: post-CCE colorectal cancer, CTC: Computed tomography colonography, This framework outlines the classification of unadjusted pCCECRC based on most plausible explanation. Category A represents a possible missed lesion following adequate prior colonic examination, and is subdivided into A1 (attributed to adequate CCE) and A2 (attributed to adequate follow-up colonoscopy, or CTC). Where multiple investigations are performed, an adequate follow-up test may supersede an incomplete or inadequately cleansed index CCE. Some patients will have an incomplete CCE and a completion flexible sigmoidoscopy, attribution is based on the lesion location, and the extent of both tests, and clinical judgement

We propose a similar attribution of most plausible explanation of pCCECRC (Fig. 1), with category A being subdivided into A1: attributed to adequate CCE, and category A2: attributed to adequate follow-up CTC/Colonoscopy. Where multiple investigations are performed, an adequate follow-up test may supersede an incomplete or inadequately cleansed index CCE.

Category A can include, for example:

- Patients who had a complete and adequately cleansed CCE where no pathology was detected. This includes lesions mischaracterised as “diminutive”.
- Patients who had a complete and adequately cleansed follow-up colonoscopy or CTC after CCE for any reason (including poor CCE prep, incomplete CCE, detected pathology on CCE). A follow-up colonoscopy that is adequate supersedes CCE, so a lesion detected on CCE but later deemed ‘false positive CCE’ (some round 1 participants mentioned this in the comments) by colonoscopy, is attributed to colonoscopy.
- Some patients will have an incomplete CCE and a completion flexible sigmoidoscopy. Attribution should be based on lesion location, the extent of each test, and clinical judgement.

*Statement 10: To facilitate categorisation of pCCECRC by most plausible explanation, we recommend CCE reporting adheres to the standard previously established by the Nyborg consensus statement on unifying terminology, reporting, and bowel preparation standards in CCE*

The Nyborg consensus statement, published in January 2025, aims to standardise reporting of CCE [24]. It provides guidance on how polyps should be described. This includes formal documentation of polyp size, location, morphology using the Paris classification where feasible, and pit pattern assessment using KUDO/NICE/JNET classification when imaging quality permits. When not enough information is available to comment on morphology of a polyp, this should be specified. When imaging quality is inadequate for pit pattern assessment, this should also be explicitly recorded. It provides recommendations on the reporting of CCE completion, and how to report signal interruptions and transit time. This includes confirmation of caecal and anal cushion visualisation, and whether the capsule was excreted. The Nyborg consensus also specifies how CCE cleansing quality should be described. Both overall bowel cleansing quality and segmental assessment of the right, transverse, and left colon should be reported using scales such as CC-CLEAR [25] or Leighton–Rex [26], alongside documentation of bowel preparation and booster regimens. These factors will allow the categorisation of pCCECRC into the most plausible explanation categories listed above.

*Statement 11: Certain aspects of PCCRC nomenclature and recommendations are not specific to colonoscopy, and their use should be extended to pCCECRC. These include:*

- *Sub-categorisation of post-test cancers into interval vs non-interval—to ensure service adherence to recommended surveillance intervals*
- *The minimum histological and molecular features data of CRC should* *be documented as per the WEO consensus statement on PCCRC*

The WEO consensus statement sub-categorises PCCRC into interval vs non-interval cancers. Interval cancers are those identified before the next screening or surveillance examination, and non-interval cancers are those identified at or after the recommended screening or surveillance interval, or where no follow-up examination was recommended [6]. These definitions should be extended to pCCECRC to ensure that appropriate screening and surveillance intervals are used by CCE services and to identify shortfalls when an interval examination is recommended but not adhered to.

Reporting the molecular and histological properties of pCCECRC allows comparison with PCCRC and identification of tumour factors that contribute to the pCCECRC rate. These include features such as tumour location, size, morphology, histologic type and grade, pathological staging (pT/pN), lymphovascular and perineural invasion, tumour budding, tumour deposits, and resection margin status. Molecular assessment should include microsatellite instability (MSI) status in all cases, as this helps identify mismatch repair deficiency and Lynch syndrome. BRAF, KRAS/NRAS, and other targetable molecular alterations should also be assessed when clinically appropriate. These molecular and histological features are summarised in Table 4.

**Table 4:** Minimum histological and molecular data to document for pCCECRC cases

Domain	Variable
Tumour pathology	Tumour location
	Tumour size
	Tumour morphology
	Histological type
	Grade
	pT/pN stage
Adverse pathological features	Lymphovascular invasion
	Perineural invasion
	Tumour budding
	Tumour deposits
	Margin status
Molecular features	MSI/MMR status in all cases
	BRAF and KRAS/NRAS where clinically appropriate
	other targetable alterations where relevant

## Discussion

In this international Delphi process, we developed an expert consensus framework for defining, reporting, and attributing pCCECRC, including the distinction between unadjusted and adjusted pCCECRC, to differentiate service evaluation from test evaluation. This work addresses a gap in QA for CCE services, which, although expanding over the past few years, lack standardised outcome-based performance metrics.

This statement is not intended to be a departure from the WEO consensus statement on PCCRC and PICRC. It was conceived to avoid confusion on what needs to be included in the pCCECRC calculation. Some, for example, may only include complete and adequately cleansed tests. Others may include incomplete tests where pathology was detected, occasionally referred to as a “definitive” test in CCE literature. Following discussion amongst the core group, the need for separate definitions for service and test evaluations became apparent. A central feature of this consensus is the distinction between unadjusted and adjusted pCCECRC rates, reflecting the dual role of CCE as both a diagnostic test and a component of a broader diagnostic pathway. The unadjusted pCCECRC rate captures service-level performance across the entire pathway, including the adequacy and timeliness of downstream investigations. On the other hand, the adjusted pCCECRC rate focuses on diagnostic accuracy in complete and adequately cleansed examinations, isolating test performance from pathway-related factors. This distinction recognises the inherent differences between CCE and colonoscopy, particularly with respect to completion rates and reliance on follow-up investigations.

Where feasible, the panel supported the alignment of pCCECRC terminology with that of PCCRC. This consistency allows comparison between CCE and colonoscopy services within healthcare systems and supports shared approaches to audit and quality improvement. However, the CCE pathway may include incomplete examinations or multiple investigations, which complicates attribution of post-test cancers. Applying the “most plausible explanation” framework offers a structured means of classification that accommodates this complexity. Standardised attribution of aetiology is also useful during medico-legal review, where clear terminology can distinguish between diagnostic test limitations and pathway/service delays, reducing ambiguity during case reviews.

This statement was designed to answer a specific question, namely how pCCECRC should be reported and classified. It is intended to be used in conjunction with established PCCRC methodology as defined by the WEO consensus statement and with existing CCE reporting standards that define test adequacy previously established in articles such as the Nyborg consensus statement. The proposed framework remains provisional until validated using real-world service data.

Implementation of this framework as part of a QA process will add administrative burden to services, and may not be realistically achievable for all providers, particularly within fragmented healthcare systems or when data access is limited. However, as a relatively new diagnostic test, it is important to monitor CCE performance against other diagnostic modalities, and to identify any inherent pathway challenges leading to pCCECRC. Robust prospective recording of CCE and CRC in registries with reliable data linkage is the best way to identify cases. Where the diagnostic pathway is undertaken across multiple providers, every reasonable effort should be made to ensure cross-service data linkage and clear governance arrangements so that pCCECRC is not under-reported.

As a Delphi process, and like most expert consensus statements, this statement reflects the experience of its panellists, most of whom practice in Europe (mostly UK based). whilst this is the result of the recruitment strategy to recruit experts involved in established CCE services, it may not capture the limitations and challenges that will be faced with further expansion of CCE internationally. Additionally, this geographic distribution of panellists may introduce pathway bias, favouring service models that may not be applicable in other healthcare settings. Given the specialised nature of CCE, panellist recruitment intentionally focussed on CCE experts, and excluded other non-CCE stakeholders input from radiology, pathology, QA, and patient representative perspectives. This limits the breadth of viewpoints and may be relevant when this methodology is validated using real-world service data.

The essential criteria for participation aimed to balance expert recruitment without being too restrictive and inadvertently excluding individuals with substantial real-world CCE experience and relevant research output. Although the “emerging experts” criterion may have introduced some subjectivity, this was mitigated through targeted recruitment via the iCARE group and direct invitation of CCE experts. Panellist characteristics in Table 2 suggest this balance may have been reasonably achieved.

Because there was little evidence on which to base the statement items, panel expertise in CCE was essential. During the systematic review, title and abstract screening was undertaken initially by a single author. This could have increased the risk of missed eligible studies and selection bias. However, this approach was used to support timely completion of the review process and is not expected to have a significant impact on the consensus statements given the mitigating factors of panel expertise and the lack of available evidence on pCCECRC.

The lack of service-level pCCECRC data availability makes this statement difficult to validate, but the similarity with PCCRC and PICRC provides reassurance that it will likely be applicable in clinical practice.

## Conclusion

This Delphi consensus statement proposes standardised definitions and reporting recommendations for pCCECRC based on expert opinion. Separate unadjusted and adjusted pCCECRC rates are intended to support service-level quality assurance and test-specific evaluation of CCE. The proposed framework should be regarded as provisional and should be refined through application to real-world service-level datasets.

## Supplementary Information

Below is the link to the electronic supplementary material.

Supplementary file1 (DOCX 51 KB)
