# Correction: Defining post–colon capsule endoscopy colorectal cancer (pCCECRC)—an International Capsule Endoscopy Research (iCARE) group consensus statement

**DOI:** 10.1007/s00464-026-13280-9

**Published:** 2026-09-08

**Authors:** Hussain Ibrahim, Ian Lei, Peter Murchie, Anastasios Koulaouzidis, Angus Watson, the iCARE consensus collaborative, James Turvill, Sunil Dolwani, Stephan Haas, Artur Nemeth, Ervin Toth, Begoña González Suárez, Lucian Negreanu, Aileen McKinley, Niels Qvist, Foong Way David Tai, Deirdre McNamara, Stephen McSorley, Ioanna Parisi, Faidon-Marios Laskaratos, Ed Seward, Cristiano Spada, Reena Sidhu, Martin Keuchel, John Plevris, Ulrik Deding, Bruno Rosa, Eimear Gibbons, Ruari Jardine, Campbell Macleod, Gemma Mcinally, Gabriele Wurm Johansson, Sergio Cadoni, Renato Cannizzaro, Anirudh Pramod Bhandare, Amit Chattree, Victoria Fawcett, Alexander Robetson, Wojciech Marlicz, Feliz Akyuz, Jack Winter, Fraser Maxwell

**Affiliations:** 1 University of Aberdeen, Aberdeen, UK; 2 NHS Highland, Inverness, UK; 3 University Hospital of Coventry and Warwickshire, Coventry, UK; 4 University of Southern Denmark, Odense, Denmark

**Correction to: Surgical Endoscopy (2026) 40:4548–4557** **https://doi.org/10.1007/s00464-026-12892-5**

The original online version of this article was revised to add and tag the names of the iCARE consensus collaborative group members.

The original article has been corrected.

