# Supplementary material for: Defining post–colon capsule endoscopy colorectal cancer (pCCECRC)—an International Capsule Endoscopy Research (iCARE) group consensus statement
Source: Surg Endosc. 2026 May 27;40(6):4548–57. doi: 10.1007/s00464-026-12892-5 (PMC13246844; doi:10.1007/s00464-026-12892-5)
Supplement: Supplementary file 1 — Supplementary file1 (DOCX 51 KB) [file 464_2026_12892_MOESM1_ESM.docx]

**Supplementary Material**

*Systematic review*

Search strings used:

Search 1:

1. exp Colorectal Neoplasms/ or exp Colon Neoplasms/ or exp Rectal Neoplasms/

2. (colorectal adj3 (cancer* or neoplasm* or carcinoma* or adenocarcinoma* or malignan*)).tw,kf.

3. (colon adj3 (cancer* or neoplasm* or carcinoma* or adenocarcinoma* or malignan*)).tw,kf.

4. (rectal adj3 (cancer* or neoplasm* or carcinoma* or adenocarcinoma* or malignan*)).tw,kf.

5. 1 or 2 or 3 or 4

6. exp Colonoscopy/ or exp Endoscopy, Gastrointestinal/

7. ((colonoscopy or colonoscopic or sigmoidoscopy or bowel) adj2 (scope or screening)).tw,kf.

8. 6 or 7

9. ((postcolonoscopy or post-colonoscopy) adj3 (cancer* or neoplasm*)).tw,kf.

10. (PCCRC or "interval colorectal cancer*" or "missed colorectal cancer*" or "delayed diagnosis" or "post procedure cancer*").tw,kf.

11. 9 or 10

12. 5 and 8 and 11

Search 2:

1. (Colon capsule endoscop* or colon capsule endoscopy/ or

((capsule* or videocapsule*) adj3 colon*) or

(pillcam colon* or (pill adj cam*)) or

(exp Colonoscopy/ or exp Intestine, Large/ or

(sigmoidoscop* or rectoscop* or colonoscop*)).ti,ab,tw.)

2. colon cancer.mp.

[mp = ti, ab, hw, tn, ot, dm, mf, dv, kf, fx, dq, bt, nm, ox, px, rx, an, ui, sy, ux, mx]

3. colorectal cancer.mp.

[mp = ti, ab, hw, tn, ot, dm, mf, dv, kf, fx, dq, bt, nm, ox, px, rx, an, ui, sy, ux, mx]

4. crc.mp.

[mp = ti, ab, hw, tn, ot, dm, mf, dv, kf, fx, dq, bt, nm, ox, px, rx, an, ui, sy, ux, mx]

5. cancer.mp.

[mp = ti, ab, hw, tn, ot, dm, mf, dv, kf, fx, dq, bt, nm, ox, px, rx, an, ui, sy, ux, mx]

6. 2 or 3 or 4 or 5

7. 1 and 6

Supplementary figure 1. Flowchart of search results and study selection

**Identification of studies via databases and registers**

Records removed *before screening*:

Duplicate records removed (n = 680)

Records identified from Databases (n = 2215)

**Identification**

Records screened

(n = 1535)

Records excluded**

(n = 1478)

Reports sought for retrieval

(n = 57)

Reports not retrieved

(n = 0)

**Screening**

Reports assessed for eligibility

(n = 57)

Excluded (n = 38):

Not related to PCCRC or pCCECRC methodology and attribution of aetiology

Studies included in review

(n = 19)

**Included**

^Source: Page MJ, et al. BMJ 2021;372:n71. doi: 10.1136/bmj.n71.^

Studies used for statement generation after full text review:

Wilson N, Bilal M, Westanmo A, Karna R, Gravely A, Shaukat A (2026) Postcolonoscopy Colorectal Cancer in Fecal Immunochemical Test-Positive Individuals: Prevalence, Predictors, and Root Cause Analysis in a Nationwide Cohort. Am J Gastroenterol 121:. <https://doi.org/10.14309/AJG.0000000000003635>

Harma CL, Jayawardena T, Ismail AGM, Lall V, Kumarasinghe P, De Boer B, Hemmings C, Amanuel B, Kelty E, Mirzai B, Guo BB, Allcock R, Salama M, Raftopoulos S, Yusoff I, Segarajasingam D, Erber WN, Ee H (2025) Post-colonoscopy colorectal cancer in the Western Australian population: analysis of patient, histopathological and molecular characteristics. Intern Med J 55:444–452. <https://doi.org/10.1111/IMJ.16650>

Ruiz-Rodríguez J, Román de la Fuente C, Torres Nieto MÁ, Bayo Juanas P, Ruiz Núñez I, Martínez Cuevas C, Sanjosé Crespo A, Díez Redondo P, García-Alonso FJ (2025) Uncovering missed opportunities - A root-cause analysis of post-colonoscopy colorectal cancer in a tertiary care setting. Revista espanola de enfermedades digestivas 117:730–736. <https://doi.org/10.17235/REED.2025.11320/2025>

Rasmussen SL, Pedersen L, Torp-Pedersen C, Rasmussen M, Bernstein I, Thorlacius-Ussing O (2025) Post-colonoscopy colorectal cancer and the association with endoscopic findings in the Danish colorectal cancer screening programme. BMJ Open Gastroenterol 12:. <https://doi.org/10.1136/BMJGAST-2024-001692>

1. Winter J, Clark G, Steele R, Thornton M (2025) Post-colonoscopy cancer rates in Scotland from 2012 to 2018: A population-based cohort study. Colorectal Dis 27:. https://doi.org/10.1111/CODI.17298

Kader R, Hadjinicolaou A V., Burr NE, Paul B, Ahmad OF, Lasse P, Manish C, Roland V, Danail S, Lovat LB (2025) Systematic Review and Meta-analysis: The Three-year Post-colonoscopy Colorectal Cancer Rate as per the World Endoscopy Organization Methodology. Clin Gastroenterol Hepatol 23:. <https://doi.org/10.1016/J.CGH.2024.07.039>

Turvill J, Haritakis M, Pygall S, Bryant E, Cox H, Forshaw G, Musicha C, Allgar V, Logan R, McAlindon M (2025) Multicentre Study of 10,369 Symptomatic Patients Comparing the Diagnostic Accuracy of Colon Capsule Endoscopy, Colonoscopy and CT Colonography. Aliment Pharmacol Ther. <https://doi.org/10.1111/APT.70046>

MacLeod C, Rajapaksha N, Brown C, Hudson J, Asif Z, Watson AJM, Mowat C, Cartlidge P, Gratton R, Winters J, Bell Z, Ray C, Maxwell F, McKinley A, Noble C, Collins P, Wilson L, Cruikshank N, Hendry P, Fletcher J, Weber B (2025) The ScotCap registry: An evaluation of 1000 colon capsule endoscopy procedures carried out in Scotland. Colorectal Disease 27:e17271. <https://doi.org/10.1111/CODI.17271>

MacLeod C, Oliphant R, Docherty JG, Watson AJM (2022) A colorectal cancer missed by colon capsule endoscopy: a case report. BMC Gastroenterol 22:258. <https://doi.org/10.1186/S12876-022-02332-8>

Vuik FER, Nieuwenburg SAV, Moen S, Spada C, Senore C, Hassan C, Pennazio M, Rondonotti E, Pecere S, Kuipers EJ, Spaander MCW (2021) Colon capsule endoscopy in colorectal cancer screening: A systematic review. Endoscopy 53:. <https://doi.org/10.1055/a-1308-1297>

Utano K, Katsuki S, Matsuda T, Mitsuzaki K, Fujita T, Nemoto D, Nagata K, Lefor AK, Togashi K (2020) Colon Capsule Endoscopy versus CT Colonography in Patients with Large Non-Polypoid Tumours: A Multicentre Prospective Comparative Study (4CN Study). Digestion 101:615–623. <https://doi.org/10.1159/000501609>

Pecere S, Senore C, Hassan C, Riggi E, Segnan N, Pennazio M, Sprujievnik T, Rondonotti E, Baccarin A, Quintero E, Adrian de Ganzo Z, Costamagna G, Spada C (2020) Accuracy of colon capsule endoscopy for advanced neoplasia. Gastrointest Endosc 91:. <https://doi.org/10.1016/j.gie.2019.09.041>

Kobaek-Larsen M, Kroijer R, Dyrvig AK, Buijs MM, Steele RJC, Qvist N, Baatrup G (2018) Back-to-back colon capsule endoscopy and optical colonoscopy in colorectal cancer screening individuals. Colorectal Disease 20:. <https://doi.org/10.1111/codi.13965>

Kang JHE, Evans N, Singh S, Samadder NJ, Lee JK (2021) Systematic review with meta-analysis: the prevalence of post-colonoscopy colorectal cancers using the World Endoscopy Organization nomenclature. Aliment Pharmacol Ther 54:1232–1242. <https://doi.org/10.1111/APT.16622>

Burr NE, Derbyshire E, Taylor J, Whalley S, Subramanian V, Finan PJ, Rutter MD, Valori R, Morris EJA (2019) Variation in post-colonoscopy colorectal cancer across colonoscopy providers in English National Health Service: population based cohort study. The BMJ 367:l6090. <https://doi.org/10.1136/BMJ.L6090>

Pedersen L, Valori R, Bernstein I, Lindorff-Larsen K, Green C, Torp-Pedersen C (2019) Risk of post-colonoscopy colorectal cancer in Denmark: Time trends and comparison with Sweden and the English National Health Service. Endoscopy 51:733–741. <https://doi.org/10.1055/A-0919-4803/ID/JR17391-7/BIB>

Rutter MD, Beintaris I, Valori R, Chiu HM, Corley DA, Cuatrecasas M, Dekker E, Forsberg A, Gore-Booth J, Haug U, Kaminski MF, Matsuda T, Meijer GA, Morris E, Plumb AA, Rabeneck L, Robertson DJ, Schoen RE, Singh H, Tinmouth J, Young GP, Sanduleanu S (2018) World Endoscopy Organization Consensus Statements on Post-Colonoscopy and Post-Imaging Colorectal Cancer. Gastroenterology 155:909-925.e3. <https://doi.org/10.1053/J.GASTRO.2018.05.038>

1. Yamada K, Nakamura M, Yamamura T, Maeda K, Sawada T, Mizutani Y, Ishikawa T, Furukawa K, Ohno E, Miyahara R, Kawashima H, Hotta N, Hirooka Y (2020) Clinical Factors Associated with Missing Colorectal Polyp on Colon Capsule Endoscopy. Digestion 101:316–322. https://doi.org/10.1159/000498942

Spada C, Hassan C, Bellini D, Burling D, Cappello G, Carretero C, Dekker E, Eliakim R, De Haan M, Kaminski MF, Koulaouzidis A, Laghi A, Lefere P, Mang T, Milluzzo SM, Morrin M, McNamara D, Neri E, Pecere S, Pioche M, Plumb A, Rondonotti E, Spaander MCW, Taylor S, Fernandez-Urien I, Van Hooft JE, Stoker J, Regge D (2020) Imaging alternatives to colonoscopy: CT colonography and colon capsuleEuropean Society of Gastrointestinal Endoscopy (ESGE) and European Society of Gastrointestinal and Abdominal Radiology (ESGAR) Guideline - Update 2020. Endoscopy 52:1127–1141. https://doi.org/10.1055/A-1258-4819

*Group Authors: iCARE consensus collaborators*

| **Collaborators** | **Affiliations** |
| --- | --- |
| James Turvill | York Teaching Hospital NHS Foundation Trust, York, UK |
| Sunil Dolwani | Cardiff and Vale UHB, Cardiff, UK |
| Stephan Haas | Karolinska University Hospital, Stockholm, Sweden |
| Artur Nemeth | Skåne University Hospital, Lund University, Malmö, Sweden |
| Ervin Toth | Skåne University Hospital, Lund University, Malmö, Sweden |
| Begoña González Suárez | Hospital Clínic de Barcelona, Barcelona, Spain |
| Lucian Negreanu | Spitalul Universitar de Urgenţă Bucureşti, Romania |
| Aileen McKinley | Aberdeen Royal Infirmary, Aberdeen, UK |
| Niels Qvist | Odense University Hospital, Odense, Denmark |
| Foong Way David Tai | Sheffield Teaching Hospitals NHS Foundation Trust, Sheffield, UK |
| Deirdre McNamara | Tallaght University Hospital, Dublin, Ireland |
| Stephen McSorley | Glasgow Royal Infirmary, Glasgow, UK |
| Ioanna Parisi | University College London Hospital, London, UK |
| Faidon-Marios Laskaratos | St. Mark's Hospital, London, United Kingdom |
| Ed Seward | University College London Hospital, London, UK |
| Cristiano Spada | Fondazione Policlinico Universitario Agostino Gemelli, IRCCS, Rome, Italy |
| Reena Sidhu | Sheffield Teaching Hospitals NHS Foundation Trust, Sheffield, UK |
| Martin Keuchel | Agaplesion Bethesda Krankenhaus Bergedorf, Hamburg, Germany |
| John Plevris | The Royal Infirmary of Edinburgh, Edinburgh, UK |
| Ulrik Deding | University of Southern Denmark, Odense, Denmark |
| Bruno Rosa | Hospital da Senhora da Oliveira, Guimarães, Portugal |
| Eimear Gibbons | Letterkenny University Hospital, Glencar, Ireland |
| Ruari Jardine | University of Aberdeen, Aberdeen, UK |
| Campbell Macleod | Aberdeen Royal Infirmary, Aberdeen, UK |
| Gemma Mcinally | Northampton General Hospital, Northampton, UK |
| Gabriele Wurm Johansson | Skåne University Hospital, Lund, Sweden |
| Sergio Cadoni | Presidio Ospedaliero CTO di Iglesias, Iglesias, Italy |
| Renato Cannizzaro | Centro di Riferimento Oncologico Aviano, IRCCS, Italy |
| Anirudh Pramod Bhandare | Northern Care Alliance NHS Group, Salford, UK |
| Amit Chattree | South Tyneside and Sunderland NHS Foundation Trust, UK |
| Victoria Fawcett | South Tees Hospitals NHS Foundation Trust, UK |
| Alexander Robetson | University Hospitals of Leicester NHS Trust, Leicester, UK |
| Wojciech Marlicz | Pomeranian Medical University, Poland |
| Feliz Akyuz | Istanbul University, Istanbul, Turkiye |
| Jack Winter | NHS Greater Glasgow and Clyde, UK |
| Fraser Maxwell | NHS Lanarkshire, UK |
